# Past, present, and future of screening for cervical cancer in Brazil: lessons learned?

**DOI:** 10.1590/0102-311XEN134625

**Published:** 2025-09-01

**Authors:** Arn Migowski

**Affiliations:** 1 Coordenação de Pesquisa e Inovação, Instituto Nacional de Câncer, Rio de Janeiro, Brasil.; 2 Coordenação de Ensino e Pesquisa, Instituto Nacional de Cardiologia, Rio de Janeiro, Brasil.

We are experiencing a historic transition in the control of cervical cancer in Brazil, with an imminent change in both technology and - hopefully - the organizational model of the program. In 2024, the Brazilian National Commission for the Incorporation of Health Technologies into the SUS (CONITEC, acronym in Portuguese) approved the incorporation of primary screening with HPV testing, replacing cervical cytology testing (Pap smear), and linked to the establishment of an organized, population-based program ^1^. New Brazilian cervical cancer screening guidelines were then developed, which received approval from the CONITEC in February 2025 and are only awaiting final publication ^2^. In these new guidelines, the cytology is placed in the role of a triage test following the screening of cases positive for oncogenic HPV non-16 or 18, and is not recommended for primary screening in conjunction with HPV testing, which would constitute a so-called co-test ^2^.

Therefore, this is a suitable time to consider the mistakes and successes of the last 97 years, during which we have followed the unfolding of the various phases of the cycle of this technology, including its dissemination and use as a central part of public health programs throughout the world. The publication of the initial results by Georgios Papanicolaou in 1928, followed by his article published in 1941, was decisive for the dissemination of the method ^3^. In Brazil, the Pap test was gradually established as the standard for cervical cancer screening over the following decades, with coverage in the country estimated at 1.2% of female individuals over 15 years of age in 1983, according to an article published in CSP ^4^. In the late 1990s, the Viva Woman project was launched as a pilot program in some cities, followed by rapid national expansion of the screening program, which adopted a predominantly opportunistic organizational model, formalizing the Brazilian Information System on Uterine Cervical Cancer (SISCOLO, acronym in Portuguese) - precursor to the current Brazilian Cancer Information System (SISCAN, acronym in Portuguese) - as a monitoring tool ^5^.

In the decades that followed, cervical cancer screening continued to be a public health priority in the country in official publications, such as the 2006 Pact for Life, which established an 80% screening coverage target, and the Strategic Action Plan to Tackle Chronic Noncommunicable Diseases in Brazil from 2011 to 2022. Despite this, the current cervical cancer scenario in Brazil remains worrisome. The estimated incidence of 15.38 cases per 100,000 women for Brazil in 2025 is far from the World Health Organization (WHO) target of four per 100,000 and reaches as high as 20.48 per 100,000 in the North region ^6^. From 2000 to 2021, the trend of increasing mortality rates predominated in the North and Northeast regions, even after adjusting for age ^7^.

Why, even so many decades after its implementation, has the impact of the screening program fallen short of expectations in Brazil? Various implementation issues may explain this lack of effectiveness. Some are due to the characteristics of the test itself, which requires specialized training for collection and interpretation, resulting in persistent quality issues, with poor collection quality in many municipalities and low positivity rates in various states or inflated positivity rates due to inconclusive results, such as atypical squamous cells of undetermined significance ^8^. Such problems are perpetuated by the lack of effective implementation of a quality program and the execution of tests in laboratories with low production. Other implementation barriers stem from the predominantly opportunistic organization model of the Brazilian program, in which the target population is not actively sought at the recommended frequency, and women are only tested when they visit healthcare services. This model results in low efficiency and effectiveness, with a high percentage of women over-screened at a frequency lower than recommended (triennal) and in age groups outside the target population of 25 to 64 years, while many women with eligibility criteria and greater risk remain without having a single screening episode throughout their entire lives ^5^.

The article by Ribeiro et al. ^9^ - published in this issue of CSP - indicates that, despite so many years of program implementation, screening coverage in the Brazilian Unified National Health System (SUS, acronym in Portuguese) was estimated at only 35.6% in the 2021-2023 period, according to data from the SISCAN. Although several other articles have sought to estimate this coverage, these results come at an opportune time to take stock of decades of the national cervical cancer screening program in this transitional period. The authors also estimated that coverage could reach 53.9% if age and interval recommendations were followed ^9^. A true opportunity cost that, besides not bringing additional benefits, entails unnecessary risks for women and a loss of resources that could be directed at those who would actually benefit. This underscores the inefficiency of the opportunistic model and the need for a change. Furthermore, the widespread culture of annual screening created an illusory increase in coverage for many years, artificially increasing the indicator of exams ratio in the female population, which was unfortunately used as a proxy for coverage for a long time and the main indicator for monitoring the program due to the difficulty in obtaining information on the women examined.

However, we cannot yet confirm that this is the screening coverage in the SUS, as three states with low implementation of the SISCAN were excluded, including two of the most populous in the country, one of which likely has the highest coverage in the country (São Paulo) and was not included in the final estimate ^9^. This largely reflects the existence of laboratories with their own systems and the lack of a webservice to enable the exchange of information. The fact that we still have this degree of uncertainty with regards to the calculation of a relatively basic indicator, even after decades, also offers a lesson that should be learned and applied in the future early detection program: it is more efficient to invest in interoperability between information systems than attempt to implement a single system on a national scale. Another source of the underestimation of coverage is that, in addition to the three states excluded from the calculation, eight other states had SISCAN implementation rates lower than 100% ^8^. Moreover, the study period includes 2021, when special recommendations for cancer screening were in effect due to the COVID-19 pandemic ^10^. On the other hand, the much higher coverage rates reported in surveys, such as the *Brazilian National Health Survey*, are overestimated due to including exams performed through supplementary healthcare services and because the coverage is self-reported, which likely leads to overestimation due to confusion with speculum gynecological exams without necessarily screening through cytology.

Although we still do not know the actual coverage in the country, the most important message is that it could potentially be much higher if there was at least adherence to screening interval and target-population guidelines ^9^. The increase could be even greater if we included women aged 65 and older who were screened without a referral. Despite the importance of training and raising awareness among physicians and nurses about the importance of adhering to screening guidelines, history has shown that this is insufficient to reverse the situation. After all, ministerial guidelines have existed since 1988 that recommend the first screening at age 25 years of age, followed by three-year intervals after two initial negative annual exams ^11^. Therefore, the key to effective change resides in transforming the organizational model of the program.

Another essential point is that, despite the importance of coverage, we must remember that it cannot be the only metric for evaluating a screening program. In addition to coverage and quality indicators, it is also important to remember that screening is a complex intervention and even adequate coverage and good quality are not sufficient to ensure effectiveness. Another chronic problem in the country is the high rate of losses to follow-up and the fragmentation of the care process into multiple stages ^12^, thus compromising the effectiveness of the program even for women who had access to screening. This stems from the lack of regulation and integration of the care network, which is also related to the opportunistic model.

We should not be under the illusion that the simple technological shift to molecular testing will solve these problems. However, if combined with an organized, population-based program, characteristics provided by HPV testing, such as the change to a 5-year screening interval and the possibility of self-sampling, could contribute to greater coverage ^1^ ^,^ ^2^ ^,^ ^5^. The adoption of molecular testing in the SUS should accompany the progressive implementation of the organized program throughout the country, respecting minimum essential criteria and, at the same time, avoiding the coexistence of cytological screening in the same municipalities and, consequently, the practice of co-testing ^2^. Given the persistent situation of significant regional inequality in the control of this cancer ^7^. extra care must be taken not to reinforce inequities by seeking to prioritize and foster structuring in municipalities with higher incidence and mortality rates, prioritizing the most vulnerable populations.

May the historic turning point that we are experiencing also bring a paradigm shift in the organizational aspects of the program and may the lessons learned from the successes and mistakes of last decades help us pave a successful path towards the elimination of cervical cancer in Brazil.
