# Supplementary material for: Past, present, and future of screening for cervical cancer in Brazil: lessons learned?
Source: Cad Saude Publica. 2025 Sep 1;41(8):e00134625. doi: 10.1590/0102-311XEN134625 (PMC12404305; doi:10.1590/0102-311XEN134625)
Supplement: Translation

# Translation: Passado, presente e futuro do rastreamento do câncer de colo do útero no Brasil: lições aprendidas?

**DOI:** 10.1590/0102-311XPT134625

**Authors:** Arn Migowski

**Affiliations:** 1 Coordenação de Pesquisa e Inovação, Instituto Nacional de Câncer, Rio de Janeiro, Brasil.; 2 Coordenação de Ensino e Pesquisa, Instituto Nacional de Cardiologia, Rio de Janeiro, Brasil.

Estamos vivendo um momento histórico de transição no controle do câncer do colo do útero no Brasil, com a iminência de uma mudança tanto do ponto de vista tecnológico quanto - espera-se também - do modelo organizacional do programa. Em 2024, foi aprovada, na Comissão Nacional de Incorporação de Tecnologias em Saúde no Sistema Único de Saúde (CONITEC), a incorporação do rastreamento primário com teste de HPV em substituição ao citopatológico do colo do útero e de forma atrelada à instituição de um programa organizado de base-populacional ^1^. Em seguida, foram desenvolvidas as novas diretrizes brasileiras de rastreamento do câncer do colo do útero, as quais já foram aprovadas em fevereiro de 2025 na CONITEC, aguardando apenas sua publicação final ^2^. Nestas novas diretrizes, o citopatológico apresenta-se deslocado para um papel de exame de triagem após o rastreamento de casos positivos para HPV oncogênico não 16 ou 18, não sendo recomendado para rastreamento primário em conjunto com o teste de HPV, o que configuraria o chamado coteste ^2^.

Portanto, um momento propício para uma avaliação dos erros e acertos dos últimos 97 anos, nos quais acompanhamos o desenrolar das diversas fases do ciclo dessa tecnologia, incluindo sua difusão e utilização como parte central de programa de saúde pública em todo o mundo. Desde o desenvolvimento e publicação dos resultados iniciais por Georgios Papanicolaou, em 1928, seguidos de seu artigo de 1941, que foi decisivo para a maior difusão do método ^3^. No Brasil, o citopatológico foi paulatinamente se estabelecendo como padrão para rastreamento do câncer do colo do útero ao longo das décadas seguintes, de forma que a sua cobertura no país em 1983 foi estimada em 1,2% das mulheres acima de 15 anos, em artigo publicado no segundo volume de CSP (1986) ^4^. No fim dos anos 1990, o projeto Viva Mulher foi iniciado como piloto em algumas cidades, seguido por uma rápida expansão nacional do programa de rastreamento, que adotou um modelo organizacional predominantemente oportunístico, oficializando, como ferramenta de monitoramento, o Sistema de Informação do Câncer do Colo do Útero (SISCOLO), precursor do atual Sistema de Informação do Câncer (SISCAN) ^5^.

Nas décadas que se seguiram, o rastreamento do câncer do colo do útero, continuou figurando como prioridade de saúde pública no país em publicações oficiais, como por exemplo, no Pacto Pela Vida de 2006, o qual previa uma meta de cobertura de 80% para o rastreamento e no Plano de Ações Estratégicas para o Enfrentamento das Doenças Crônicas Não Transmissíveis no Brasil de 2011-2022. A despeito disso, o cenário atual do câncer do colo do útero no Brasil, ainda preocupa. A taxa de incidência de 15,38 casos a cada 100 mil mulheres, estimada para Brasil em 2025 ainda está longe da meta da Organização Mundial da Saúde (OMS) de 4 por 100 mil, chegando a 20,48 por 100 mil na Região Norte ^6^. Do ano 2000 até 2021 predominou a tendência de aumento das taxas de mortalidade nas regiões Norte e Nordeste, mesmo após ajuste por idade ^7^.

Por que, mesmo tantas décadas depois de implantado, o impacto do programa de rastreamento ficou aquém do esperado no Brasil? Diversos problemas de implementação explicam esse déficit de efetividade. Parte deles se deve a características do próprio teste, que demanda treinamento de profissionais especializados para sua coleta e interpretação. Isso resulta em problemas persistentes de qualidade, com muitos municípios com baixa qualidade da coleta e diversos estados com baixa positividade ou com positividade inflada por resultados inconclusivos como células escamosas atípicas de significado indeterminado ^8^. Problemas esses perpetuados pela falta de implementação efetiva de um programa de qualidade e pela pulverização dos exames em laboratórios com baixa produção. Outras barreiras de implementação advêm do modelo predominantemente oportunístico da organização do programa brasileiro, no qual não há convocação ativa da população-alvo na periodicidade recomendada e mulheres são rastreadas por ocasião do comparecimento aos serviços de saúde. Esse modelo organizacional resulta em baixa eficiência e efetividade, com elevado percentual de mulheres sobrerrastreadas em periodicidade menor do que a recomendada (trienal) e em faixas etárias fora da população-alvo de 25 a 64 anos, ao mesmo tempo em que muitas mulheres com critérios de elegibilidade e maior risco permanecem sem ter um único episódio de rastreamento ao longo de suas vidas ^5^.

O artigo de Ribeiro et al. ^9^, publicado neste fascículo de CSP, indica que mesmo com tantos anos de programa a cobertura do rastreamento no Sistema Único de Saúde (SUS) foi estimada em apenas 35,6% no triênio 2021-2023, segundo dados do SISCAN. Apesar de vários outros artigos já terem buscado estimar essa cobertura, esses resultados chegam em momento oportuno para fazermos um balanço de décadas do programa nacional de rastreamento de câncer do colo do útero neste momento de transição. Os autores estimaram também, que a cobertura poderia chegar a 53,9% se as recomendações de faixa etária e periodicidade fossem seguidas ^9^. Um verdadeiro custo de oportunidade que além de não trazer benefícios adicionais, traz riscos desnecessários para as mulheres e perda de recursos que poderiam ser direcionados para quem de fato se beneficiaria. Isso reforça a pouca eficiência do modelo oportunístico e necessidade de sua mudança. Além disso, a cultura disseminada do rastreamento anual criou durante muitos anos um ilusório incremento de cobertura, inflando artificialmente o indicador de razão de exames na população feminina, que infelizmente foi usado por muito tempo como *proxy* da cobertura e principal indicador de monitoramento do programa, pela dificuldade de se ter a informação sobre mulheres examinadas.

Contudo, ainda não podemos afirmar que essa é a cobertura do rastreamento pelo SUS no Brasil, pois foram excluídos três estados com baixa implantação do SISCAN, entre eles dois dos mais populosos do país, sendo um deles provavelmente com a maior cobertura do país (São Paulo) e não contabilizado na estimativa final ^9^. Isso reflete, em grande parte, a existência de laboratórios com sistema próprio e a ausência de *webservice* que permita a troca de informações. O fato de ainda termos esse grau de incerteza sobre um cálculo de indicador relativamente básico, mesmo após décadas, traz também uma lição que deve ser aprendida e aplicada no futuro programa de detecção precoce: será mais eficiente investir em interoperabilidade entre sistemas de informação do que tentar implantar um sistema único em todo o país. Outra fonte de subestimação da cobertura é que, além dos três estados excluídos do cálculo, outros oito estados apresentaram implantação do SISCAN abaixo de 100% ^8^. Além disso, o período de estudo inclui 2021, ano em que estavam vigentes recomendações especiais para o rastreamento de câncer no contexto da pandemia de COVID-19 ^10^. Por outro lado, coberturas muito mais elevadas em inquéritos, como a *Pesquisa Nacional de Saúde*, apresentam superestimação por incluírem exames realizados na saúde suplementar e por serem autoreferidos, o que deve gerar superestimação também pela confusão com o exame ginecológico especular sem que necessariamente tenha havido rastreamento com o citopatológico.

Embora continuemos sem saber qual é a real cobertura no país, a mensagem mais importante é que ela potencialmente poderia ser muito maior se ao menos houvesse adesão às diretrizes de periodicidade e população-alvo ^9^. E esse aumento poderia ser ainda maior se incluíssemos nessas estimativas as mulheres com 65 anos ou mais que foram rastreadas sem indicação. No que pese a importância da capacitação e sensibilização de médicos e enfermeiros sobre a importância da adesão às diretrizes de rastreamento, a história já comprovou que isso é insuficiente para reverter esse quadro. Afinal, já existem, desde 1988, diretrizes ministeriais que recomendam iniciar o rastreamento a partir de 25 anos e fazê-lo em periodicidade trienal após dois exames iniciais anuais negativos ^11^. Portanto, a chave para uma mudança efetiva está na transformação do modelo organizacional do programa.

Outro ponto essencial é que, apesar da importância da cobertura, precisamos lembrar que ela não pode ser a única métrica para se avaliar um programa de rastreamento. Além dela e dos indicadores de qualidade, é necessário lembrar também que o rastreamento é uma intervenção complexa e mesmo uma cobertura adequada e boa qualidade não são suficientes para garantir a efetividade. Outro problema crônico no país é a elevada perda de seguimento e a fragmentação do processo de cuidado em múltiplas etapas ^12^, comprometendo a efetividade do programa mesmo para aquelas mulheres que tiveram acesso ao rastreamento. Isso advém da falta de regulação e integração da rede assistencial, o que também está relacionado ao modelo oportunístico.

Não devemos ter a ilusão de que a simples mudança tecnológica para os testes moleculares resolverá esses problemas. Porém, se associado a um programa organizado de base-populacional, características propiciadas pelos testes de HPV, como a mudança para a periodicidade quinquenal e a possibilidade de autocoleta, poderão contribuir para maior cobertura ^1^ ^,^ ^2^ ^,^ ^5^. A adoção dos testes moleculares no SUS deve acompanhar a implantação progressiva do programa organizado no país, respeitando critérios mínimos essenciais e, ao mesmo tempo, evitar a coexistência do rastreamento citológico nos mesmos municípios e consequentemente a prática de coteste ^2^. Diante do quadro persistente de grande desigualdade regional no controle deste câncer ^7^, deve ao mesmo tempo haver um cuidado redobrado para não se reforçar iniquidades buscando-se priorizar e fomentar a estruturação em municípios com maior incidência e mortalidade, priorizando o alcance das populações mais vulneráveis.

Que o ponto de inflexão histórica que estamos vivendo traga uma mudança paradigmática também no aspecto organizacional do programa e que as lições advindas dos acertos e erros das últimas décadas possam nos ajudar a pavimentar um caminho de sucesso rumo à eliminação do câncer do colo do útero no Brasil.

# Translation: Pasado, presente y futuro del cribado del cáncer de cuello uterino en Brasil: ¿lecciones aprendidas?

**DOI:** 10.1590/0102-311XES134625

**Authors:** Arn Migowski

**Affiliations:** 1 Coordenação de Pesquisa e Inovação, Instituto Nacional de Câncer, Rio de Janeiro, Brasil.; 2 Coordenação de Ensino e Pesquisa, Instituto Nacional de Cardiologia, Rio de Janeiro, Brasil.

Vivimos un momento histórico de transición en el control del cáncer de cuello uterino en Brasil, con el inminente cambio tanto desde el punto de vista tecnológico como -esperemos- en el modelo organizativo del programa. En el 2024, se aprobó, en la Comisión Nacional de Incorporación de Tecnologías en Salud en el Sistema Único de Salud (CONITEC, por su sigla en portugués), la incorporación del cribado primario con prueba de VPH en sustitución de la citología del cuello uterino, y de forma vinculada a la institución de un programa organizado de base poblacional ^1^. A continuación, se desarrollaron las nuevas directrices brasileñas para el cribado del cáncer de cuello uterino, las cuales ya se aprobaron en febrero del 2025 en CONITEC, en espera de su publicación final ^2^. Bajo estas nuevas directrices, la citología se traslada a un papel de examen de detección tras el cribado de casos positivos para el VPH oncogénico no 16 o 18, y no se recomienda para el cribado primario junto con la prueba del VPH, lo que configuraría el llamado co-test ^2^.

Por lo tanto, este es un buen momento para evaluar los fracasos y éxitos de los últimos 97 años, durante los cuales hemos observado el desarrollo de las distintas etapas del ciclo de esta tecnología, que incluyen su difusión y uso como parte central de los programas de salud pública en todo el mundo. Desde el desarrollo y la publicación de los resultados iniciales por Georgios Papanicolaou en 1928, seguido de su artículo de 1941, que fue decisivo para la mayor difusión del método ^3^. En Brasil, la citología se fue consolidando gradualmente como el estándar para el cribado del cáncer de cuello uterino a lo largo de las décadas siguientes, de modo que su cobertura en el país en 1983 se estimó en 1,2% de las mujeres mayores de 15 años, en un artículo publicado en CSP ^4^. A finales de la década de 1990, se inició el proyecto Viva Mulher como piloto en algunas ciudades, seguido de una rápida expansión nacional del programa de cribado, que adoptó un modelo organizativo predominantemente oportunista, oficializando, como herramienta de monitoreo, el Sistema de Información de Cáncer de Cuello Uterino (SISCOLO, por su sigla en portugués), precursor del actual Sistema de Información de Cáncer (SISCAN, por su sigla en portugués) ^5^.

En las décadas siguientes, el cribado del cáncer de cuello uterino siguió siendo una prioridad de salud pública en el país en publicaciones oficiales, como, por ejemplo, el Pacto por la Vida del 2006, que estableció una meta de cobertura del 80% para el cribado, y el Plan de Acciones Estratégicas para el Enfrentamiento de las Enfermedades Crónicas No Transmisibles en Brasil 2011-2022. A pesar de ello, la situación actual del cáncer de cuello uterino en Brasil sigue siendo preocupante. La tasa de incidencia de 15,38 casos por cada 100.000 mujeres, estimada para Brasil en el 2025, aún está lejos de la meta de la Organización Mundial de la Salud (OMS) de 4 por cada 100.000, y alcanza 20,48 por 100.000 en la Región Norte ^6^. Del 2000 al 2021, predominó la tendencia de aumento de las tasas de mortalidad en las regiones Norte y Nordeste, incluso después del ajuste por edad ^7^.

¿Por qué, incluso tantas décadas después de su implementación, el impacto del programa de cribado no ha cumplido las expectativas en Brasil? Diversos problemas de implementación explican esta falta de efectividad. Parte de ellos se debe a las características de la propia prueba, que requiere capacitación de profesionales especializados para su toma e interpretación. Esto da lugar a problemas de calidad persistentes, con muchos municipios con baja calidad de la toma y varios estados con baja positividad o positividad inflada por resultados no concluyentes, como células escamosas atípicas de significado indeterminado ^8^. Estos problemas se perpetúan debido a la falta de implementación efectiva de un programa de calidad y a la dispersión de pruebas en laboratorios con baja producción. Otras barreras de implementación surgen del modelo predominantemente oportunista de organización del programa brasileño, en el que no se convoca de forma activa a la población objetivo con la frecuencia recomendada, y las mujeres son examinadas cuando asisten a los servicios de salud. Este modelo organizativo resulta en baja eficiencia y efectividad, con un elevado porcentaje de mujeres sobrecribadas en una periodicidad menor que la recomendada (trienal) y en rangos de edad fuera de la población objetivo de 25 a 64 años, a la vez que muchas mujeres con criterios de elegibilidad y mayor riesgo permanecen sin tener un solo episodio de cribado a lo largo de su vida ^5^.

El artículo de Ribeiro et al. ^9^, publicado en este número de CSP, indica que incluso con tantos años de programa, la cobertura de cribado en el Sistema Único de Salud (SUS) se estimó en solo el 35,6% en el trienio 2021-2023, según datos de SISCAN. Aunque varios otros artículos ya han buscado estimar esta cobertura, estos resultados llegan en un momento oportuno para hacer un balance de décadas del programa nacional de cribado del cáncer de cuello uterino en este momento de transición. Los autores también estimaron que la cobertura podría llegar al 53,9% si se siguieran las recomendaciones de rango de edad y periodicidad ^9^. Un verdadero costo de oportunidad que, además de no aportar beneficios adicionales, implica riesgos innecesarios para las mujeres y una pérdida de recursos que podrían dirigirse a quienes realmente se beneficiarían. Esto refuerza la baja eficiencia del modelo oportunista y la necesidad de cambiarlo. Además, la cultura difundida del cribado anual creó durante muchos años un incremento ilusorio de cobertura, aumentando artificialmente el indicador de razón de exámenes en la población femenina, que lamentablemente se utilizó durante mucho tiempo como un proxy de la cobertura y principal indicador de monitoreo del programa, debido a la dificultad de obtener información sobre las mujeres examinadas.

Sin embargo, aún no podemos afirmar que esta es la cobertura de cribado por el SUS en Brasil, ya que se excluyeron tres estados con baja implementación del SISCAN, entre ellos dos de los más poblados del país, de los cuales uno probablemente tiene la mayor cobertura del país (São Paulo) y no se contabilizó en la estimación final ^9^. Esto refleja, en gran medida, la existencia de laboratorios con su propio sistema y la ausencia de servicios *web* que permita el intercambio de información. El hecho de que aún tengamos este grado de incertidumbre sobre un cálculo de indicador relativamente básico, incluso después de décadas, también trae consigo una lección que debe aprenderse y aplicarse en el futuro programa de detección temprana: será más eficiente invertir en interoperabilidad entre sistemas de información que intentar implementar un sistema único en todo el país. Otra fuente de subestimación de la cobertura es que, además de los tres estados excluidos del cálculo, otros ocho estados tuvieron una implementación del SISCAN por debajo del 100% ^8^. Además, el periodo de estudio incluye el 2021, año en el que estuvieron vigentes recomendaciones especiales para el cribado del cáncer en el contexto de la pandemia ^10^. Por otra parte, coberturas mucho mayores en encuestas, como la *Encuesta Nacional de Salud*, presentan sobreestimación por incluir exámenes realizados en salud complementaria y por ser autorreportados, lo que también debería generar sobreestimación por confusión con el examen ginecológico con espéculo sin necesariamente haber tenido cribado con la citología.

Aunque seguimos sin saber cuál es la cobertura real en el país, el mensaje más importante es que potencialmente podría ser mucho mayor si al menos hubiera adhesión a las directrices de periodicidad y población objetivo ^9^. Y este aumento podría ser aún mayor si incluyéramos en estas estimaciones a las mujeres de 65 años o más que se sometieron a pruebas de cribado sin indicación alguna. A pesar de la importancia de capacitar y sensibilizar a los médicos y enfermeros sobre la importancia de la adhesión a las directrices de cribado, la historia ya ha demostrado que esto no es suficiente para revertir esta situación. Al fin y al cabo, ya existen desde 1988 directrices ministeriales que recomiendan comenzar el cribado a partir de los 25 años y realizarlo cada tres años después de dos exámenes anuales iniciales negativos ^11^. Por lo tanto, la clave para un cambio efectivo radica en transformar el modelo organizativo del programa.

Otro punto clave es que, a pesar de la importancia de la cobertura, debemos recordar que no puede ser la única métrica para evaluar un programa de cribado. Además de esta y de los indicadores de calidad, también es importante recordar que el cribado es una intervención compleja e incluso una cobertura adecuada y una buena calidad no son suficientes para asegurar su efectividad. Otro problema crónico en el país es la elevada pérdida de seguimiento y la fragmentación del proceso de cuidado en múltiples etapas ^12^, lo que compromete la efectividad del programa incluso para aquellas mujeres que tuvieron acceso al cribado. Esto surge de la falta de regulación e integración de la red asistencial, lo que también está relacionado con el modelo oportunista.

No debemos hacernos la ilusión de que simplemente cambiar la tecnología hacia las pruebas moleculares resolverá estos problemas. Sin embargo, si se asocia a un programa organizado de base poblacional, las características que aportan las pruebas de VPH, como el cambio a una frecuencia quinquenal y la posibilidad de autorrecolección, podrán contribuir a una mayor cobertura ^1^ ^,^ ^2^ ^,^ ^5^. La adopción de pruebas moleculares en el SUS debe acompañar la implementación progresiva del programa organizado en el país, respetando criterios mínimos esenciales y, a la vez, evitando la coexistencia de cribado citológico en los mismos municipios y, como consecuencia, la práctica de co-test ^2^. Dada la persistente situación de gran desigualdad regional en el control de este cáncer ^7^, a la vez, se debe tener especial cuidado de no reforzar las inequidades al buscar priorizar y promover la estructuración en municipios con mayor incidencia y mortalidad, priorizando llegar a las poblaciones más vulnerables.

Que el punto de inflexión histórico que estamos viviendo traiga consigo un cambio paradigmático también en el aspecto organizativo del programa, y que las lecciones aprendidas de los éxitos y fracasos de las últimas décadas puedan ayudarnos a construir un camino exitoso hacia la eliminación del cáncer de cuello uterino en Brasil.
